# Associations between health anxiety, eHealth literacy and self-reported health: A cross-sectional study

**DOI:** 10.1590/1980-220X-REEUSP-2024-0160en

**Published:** 2024-11-29

**Authors:** Jie Chen, Hua Tian

**Affiliations:** 1 Xinyang Normal University, School of Marxism, Xinyang, China.; 2 Xinyang Normal University, College of Life Science, Xinyang, China.

**Keywords:** Health Literacy, Mental Health, Gender Identity, Students, Health Education, Letramento em Saúde, Saúde Mental, Identidade de Gênero, Estudantes, Educação em Saúde, Alfabetización en Salud, Salud Mental, Identidad de Género, Estudiantes, Educación en Salud

## Abstract

**Objective:**

To explore the associations and gender differences between health anxiety, eHealth literacy and self-reported health in Chinese university students.

**Methods:**

1,205 university students aged 18–22 years were voluntarily recruited to respond to an online self-report questionnaire.

**Results:**

The severity level of health anxiety among university students was ranked as lifestyle anxiety, psychological anxiety, appearance anxiety, physical anxiety. There were significant gender differences in appearance anxiety, and yet no in eHealth literacy of university students. eHealth literacy was positively associated with self-reported health; health anxiety was negatively associated with self-reported health. Female’s eHealth literacy, lifestyle, psychological and physical anxiety, and male’s eHealth literacy, appearance anxiety significantly impacted on their self-reported health.

**Conclusion:**

The lower eHealth literacy or the more health anxiety, the worse their self-reported health. The findings underscored the importance for university students to improve eHealth literacy and reduce health anxiety. Appropriate interventions with gender differences were urgently needed.

## RESUMO

**Objetivo:**

Explorar as associações e diferenças de gênero entre ansiedade em saúde, alfabetização em e-Saúde e saúde autorrelatada em estudantes universitários chineses.

**Métodos:**

1.205 estudantes universitários com idades entre 18 e 22 anos foram recrutados voluntariamente para um questionário online autorrelatado.

**Resultados:**

O nível de gravidade da ansiedade em saúde entre estudantes universitários foi classificado como ansiedade de estilo de vida, ansiedade psicológica, ansiedade de aparência, ansiedade física. Houve diferenças significativas de gênero na ansiedade de aparência, mas nenhuma na alfabetização em e-Saúde de estudantes universitários. A alfabetização em e-Saúde foi positivamente associada à saúde autorrelatada; a ansiedade em saúde foi negativamente associada à saúde autorrelatada. A alfabetização em e-Saúde, estilo de vida, ansiedade psicológica e física das mulheres e a alfabetização em e-Saúde, ansiedade de aparência dos homens impactaram significativamente em sua saúde autorrelatada.

**Conclusão:**

Quanto menor a alfabetização em e-Saúde ou maior a ansiedade em saúde, pior sua saúde autorrelatada. As descobertas ressaltaram a importância de os estudantes universitários melhorarem a alfabetização em e-Saúde e reduzirem a ansiedade em saúde. Intervenções apropriadas com diferenças de gênero eram urgentemente necessárias.

## RESUMEN

**Objetivo:**

Explorar las asociaciones y diferencias de género entre ansiedad en salud, alfabetización en cibersalud y salud autoinformada en estudiantes universitarios chinos.

**Métodos:**

Se reclutaron voluntariamente 1.205 estudiantes universitarios, con edad comprendida entre 18 y 22 años, para un cuestionario online autoinformado.

**Resultados:**

El nivel de gravedad de ansiedad en salud entre los estudiantes universitarios fue clasificado como ansiedad de estilo de vida, ansiedad psicológica, ansiedad de apariencia física. Se encontraron diferencias significativas de género en la ansiedad de apariencia, pero ninguna en la alfabetización en cibersalud. La alfabetización en cibersalud estuvo asociada positivamente a la salud autoinformada; la ansiedad en salud estuvo asociada negativamente a la salud autoinformada. La alfabetización en cibersalud, estilo de vida, ansiedad psicológica y física de las mujeres y la alfabetización en cibersalud, la ansiedad de apariencia de los hombres impactaron significativamente en su salud autoinformada.

**Conclusión:**

A menos alfabetización en cibersalud o más ansiedad en salud, se desmejora la salud autoinformada. Los hallazgos resaltan la importancia de que los estudiantes universitarios mejoren la alfabetización en cibersalud para así disminuir la ansiedad en salud. Son necesarias intervenciones urgentes y apropiadas con diferencias de género.

## INTRODUCTION

The World Health Organization’s World Mental Health Surveys showed that 20.3% of university students in 21 countries have mental disorders^(1)^. University students face numerous health problems in their daily lives, especially mental health issues, such as health anxiety, depression, inferiority complex, interpersonal sensitivity, and other frequent occurrences that have generated global consensus^(2)^. Normal attention to health can sometimes turn into a persistent and excessive fear of serious illness. Svestkova et al.^(3)^ defined health anxiety as distress or fear related to one’s body. Health anxiety represents excessive contemplation of illness, excessive concern for physical health, persistent fear of illness, misinterpreting bodily sensations as symptoms of severe illness, and reporting symptoms without sufficient physical pathology. Health anxiety can lead to both psychological and physical symptoms, which are often misunderstood as evidence of organic diseases. It ranges from mild anxiety to severe or persistent anxiety^(4)^. Health anxiety is a serious and costly public health issue^(5)^; if left untreated, it may become chronic. Studies indicate that university students use the Internet more frequently than any other group and often seek online health information^(6)^. The daily lives of college students are filled with health anxiety, and the boundary between “health” and “disease” is gradually blurred. Many diseases are merely self-created “suspected diseases,” rather than genuine psychological or physiological problems^(7)^.

University students are particularly prone to sleep disorders, eating disorders, anxiety and depression, or chronic diseases^(8)^. Health anxiety is associated with an increased risk of developing various chronic diseases^(9)^. Compared to male students, female university students typically exhibit lower adaptability to university life, as well as higher concerns and physiological sensitivity^(10)^. In this study, health anxiety was defined as the anxiety or distress caused by an individual’s or their family members’ unreasonable lifestyle, physical weakness or illness, depression, anxiety, emotional instability, overpressure, as well as dissatisfaction with their appearance or body shapes, including lifestyle anxiety, psychological anxiety, physical anxiety, and appearance anxiety.

Electronic health literacy, as a part of a rational cognitive belief system, can help individuals correctly and objectively understand and comprehend things. Individuals with a certain level of eHealth literacy are less likely to blindly believe that their health condition is threatened and not easily develop negative emotions such as health anxiety when facing massive online health information^(11)^. EHealth literacy refers to the ability of individuals to seek, search, understand and evaluate health information from electronic resources, and apply this knowledge to solve or handle health problems^(12)^. EHealth literacy has a significant and direct positive impact on the mental health of university students^(13)^. Although university students have the skills to access online information, they often lack the corresponding medical and health knowledge to determine the authenticity of health information^(14)^.

Could we speculate that the lower eHealth literacy of university students, the more health anxiety, thus, the worse self-reported health? Few relevant studies were found concerning this matter. Therefore, this study used online questionnaire survey and aimed to explore the associations and gender differences among health anxiety, eHealth literacy and self-reported health in Chinese university students. The findings can provide reference for intervention training of eHealth literacy and health anxiety among university and college students based on gender differences to improve overall health levels.

## METHOD

### Study Design

Cross-sectional quantitative study.

### Setting

The study was conducted at a university in the city of Xinyang City, Henan Province, China-Xinyang Normal University. Xinyang Normal University is a full-time general higher education institution sponsored by the People’s Government of Henan Province and supervised by the Education Department of Henan Province.

### Sample Selecton Criteria

University students were recruited from Xinyang Normal University, who were voluntarily aware that their participation would be anonymous when completing the online self-report questionnaire. They signed a Free Informed Consent form, which included information of health anxiety, eHealth literacy, self-reported health, as well as sociodemographic questions (residence, age, education, and parental education level). If the answer for the questionnaire was incomplete or there was no answer, the respondent would be excluded. However, statistics found that all university students (1,205) had satisfactorily completed the questionnaire (response rate: 100%).

### Instrument and Study Period for Data Collection

Questionnaire used in this study included eHealth literacy scale (Chart 1), questions of health anxiety^(15)^ (Chart 2) and self-reported health (One item: “How well do you currently feel with respect to your health status?”), which were created in Wenjunxing software (https://www.wjx.cn/). Wenjuanxing software is a professional online survey, examination, evaluation, and voting platform that focuses on providing users with a series of services such as powerful and user-friendly online questionnaire design, data collection, custom reports, and survey result analysis.

**Chart 1:** eHealth literacy scale – Xinyang, Henan, China, 2024.

Items	
Functional eHealth literacy	
1	I cannot understand the symbols (such as BMI, Body Mass Index) and wording about health information.
2	I find the online health information difficult to understand.
3	I find the mathematical formulas provided in online health information difficult to calculate. (e.g., the algorithm of calorie consumption, BMI).
Interactive eHealth literacy	
4	I can locate health information efficiently through search engines.
5	I pay attention to and obtain new knowledge about online health information.
6	I know how to get what I need from online health information.
7	I understand the online health information I have obtained.
Critical eHealth literacy	
8	I will think about whether the online health information applies to my situation.
9	I try to find different sources to verify the credibility of health information.
10	I evaluate the validity and reliability of online health information.
11	I will browse various discussions and plan or action that is good for health.
12	When I have questions or doubts about online health information, I use other channels to verify the information.
5-point Likert scale: Ranging from 1 for “strongly disagree” to 5 for “strongly agree.” Each item score varies between 1 and 5. The total score varies between 12 and 60. The higher score indicates the higher eHealth literacy level.	

Note. The eHealth literacy scale is cited from the literature^(16)^.

**Chart 2:** Questionnaire of health anxiety – Xinyang, Henan, China, 2024.

Characteristics	Scores
Lifestyle anxiety	The sum of the following five health anxiety scores
Lack of exercise	0 = No, 1 = Yes
Insufficient sleep	0 = No, 1 = Yes
Unreasonable diet	0 = No, 1 = Yes
Smoking or second-hand smoke	0 = No, 1 = Yes
Excessive drinking	0 = No, 1 = Yes
Psychological anxiety	The sum of the following four health anxiety scores
Emotional instability	0 = No, 1 = Yes
Anxious	0 = No, 1 = Yes
Overpressure	0 = No, 1 = Yes
Depression	0 = No, 1 = Yes
Physical anxiety	The sum of the following five health anxiety scores
Physical weakness	0 = No, 1 = Yes
Family illness	0 = No, 1 = Yes
Suffering from chronic diseases	0 = No, 1 = Yes
Suffering from infectious diseases	0 = No, 1 = Yes
Suffering from critical illness	0 = No, 1 = Yes
Appearance anxiety	The sum of the following three health anxiety scores
Skin problem	0 = No, 1 = Yes
Alopecia	0 = No, 1 = Yes
Obesity	0 = No, 1 = Yes

Note. There were multiple choices for health anxiety in the past two weeks, including four dimensions.

First, questionnaire was created using Wenjuanxing software. Then, the questionnaire link was sent to the WeChat groups of Xinyang Normal university students so that they could respond. The questionnaire is anonymous and voluntary. Voluntary participant university students can freely and independently fill out questionnaires on their computers or smartphones. WeChat was chosen because users are verified without virtually possibilities to incur in fake profiles. There is no reward for participants.

The eHealth literacy questionnaire used an eHealth literacy scale designed by Chiang et al ^(16)^. The scale consists of three levels: functional (three items), interactive (four items), and critical (five items). Functional eHealth literacy evaluates individuals’ reading and writing skills, as well as their understanding of basic online health information. Interactive eHealth literacy evaluates individuals’ skills and abilities which can be used to access information from various forms of social online environments. Critical eHealth literacy evaluates individuals’ abilities and skills which can be used to critically evaluate online health information and utilize it to make informed health decisions. The 12-item eHealth literacy scale uses a 5-point Likert scale: ranging from 1 for “strongly disagree” to 5 for “strongly agree.” Each item score varies between 1 and 5. The total score varies between 12 and 60. The higher score indicates the higher eHealth literacy level is. The Kaiser-Meyer-Olkin was 0.907, and Cronbach’s Alpha was 0.793, showing good internal validity and reliability.

The questionnaire of health anxiety contains four dimensions, including lifestyle anxiety, psychological anxiety, physical anxiety, and appearance anxiety. Each containing different questions (0 = No, 1 = Yes). The total score was used to evaluate one dimension. Higher scores indicate more health anxiety.

The self-reported health status was measured by one item: “How well do you currently feel with respect to your health status?” (5 = excellent, 4 = good, 3 = neutral, 2 = poor, 1 = very poor).

The questionnaire investigation was conducted from March to May 2023.

### Data Analysis

Self-reported health was the dependent variable, defined as “5 = excellent, 4 = good, 3 = neutral, 2 = poor, 1 = very poor”. Thus, ordinal logistic regression was used to analyze the associations and gender differences among eHealth literacy, health anxiety and self-reported health for Chinese university students. SPSS v20 (IBM, Armonk, NY, United States) was used for descriptive statistics (mean, standard deviation [SD], frequency, and percentage), chi-squared test, t-test and ordinal logistic regression were used for analysis.

### Ethical Aspects

This study was performed in compliance with the Helsinki Declaration guidelines. All procedures relevant to study participants were approved by Xinyang Normal University ethics committee (XFEC-2023-025). Each voluntary participant was informed of the study objective and context and provided their informed consent regarding privacy and information management policies. University students whose survey results are negative in the investigation will receive more health care from psychology professors and school nurses to reduce health anxiety.

## RESULTS

### Demographic Characteristics

Out of the 1,205 university student participants (Table 1), 57.82% were male and 65.79% were females from rural areas. 48.82% males major was in science. For female university students, 43.96% and 43.36% majored in humanities and science, respectively. Comparing males and females, there were more females majored in humanities than males. Parents of males and females generally had low level of education, e.g., middle school (males: 57.82%, females: 68.01%). Self-reported good health status of university students, e.g., males: 40.28% were good and 28.44% were excellent; females: 47.99% were good and 34.81% were neutral. In summary, there were significant gender differences in all five variables.

**Table 1:** Characteristics of the participants (n = 1,205) – Xinyang, Henan, China, 2024.

Characteristics	Frequency (%)				P
	Male (n = 211, %)		Female (n = 994, %)		
Residence					0.028
Urban	89	42.18	340	34.21	
Rural	122	57.82	654	65.79	
Age (years)					<0.001
Average	19.39 ± 1.143		19.01 ± 0.933		
Major					<0.001
Humanities	52	24.64	437	43.96	
Science	103	48.82	431	43.36	
Engineering	56	26.54	126	12.68	
Parental education level					0.005
PhD/Master	5	2.37	8	0.80	
Undergraduate major/ Higher education	84	39.81	310	31.19	
Middle school	122	57.82	676	68.01	
Self-reported health					<0.001
Excellent	60	28.44	156	15.69	
Good	85	40.28	477	47.99	
Neutral	59	27.96	346	34.81	
Poor	5	2.37	14	1.41	
Very poor	2	0.95	1	0.10	

Note. P-values were yielded by chi-squared test and t-test.

### Gender Differences in Health Anxiety

In this study, health anxiety was divided into four dimensions, namely lifestyle anxiety, psychological anxiety, physical anxiety, and appearance anxiety. A total of 68% of university students had three or more health anxieties. From Table 2, there were significant gender differences in appearance anxiety (P < 0.001).

**Table 2:** Gender differences in health anxiety (n = 1,205) – Xinyang, Henan, China, 2024.

Characteristics	Total frequency (%)		Male (%)		Female (%)		P	
Lifestyle anxiety			1.68 ± 1.134		1.75 ± 1.034		t	0.362
Lack of exercise	810	67.22	113	53.55	697	70.12	χ^2^	< 0.001
Insufficient sleep	740	61.41	129	61.14	611	61.47	χ^2^	0.928
Unreasonable diet	496	41.16	88	41.71	408	41.05	χ^2^	0.860
Smoking or second-hand smoke	31	2.57	13	6.16	18	1.81	χ^2^	< 0.001
Excessive drinking	17	1.41	11	5.21	6	0.60	χ^2^	< 0.001
Psychological anxiety			0.99 ± 1.246		1.15 ± 1.221		t	0.079
Emotional instability	498	41.33	71	33.65	427	42.96	χ^2^	0.013
Anxiousness	444	36.85	60	28.44	384	38.63	χ^2^	0.005
Overpressure	338	28.05	58	27.49	280	28.17	χ^2^	0.842
Depression	76	6.31	20	9.48	56	5.63	χ^2^	0.037
Appearance anxiety			0.62 ± 0.872		1.05 ± 1.006		t	< 0.001
Skin problem	460	38.17	56	26.54	404	40.64	χ^2^	< 0.001
Alopecia	428	35.52	36	17.06	392	39.44	χ^2^	< 0.001
Obesity	288	23.90	39	18.48	249	25.05	χ^2^	0.042
Physical anxiety			0.31 ± 0.674		0.35 ± 0.565		t	0.440
Physical weakness	310	25.73	43	20.38	267	26.86	χ^2^	0.050
Family illness	72	5.98	10	4.74	62	6.24	χ^2^	0.404
Suffering from chronic diseases	16	1.33	8	3.79	8	0.80	χ^2^	0.001
Suffering from infectious diseases	9	0.75	2	0.95	7	0.70	χ^2^	0.709
Suffering from critical illness	4	0.33	3	1.42	1	0.10	χ^2^	0.002

Note. There were multiple choices for health anxiety in the past two weeks, including four dimensions. Data were expressed as frequency (%) and mean. P-values were yielded by chi-squared test and t-test.

For lifestyle anxiety, there were significant gender differences in lack of exercise (P < 0.001) and smoking or second-hand smoke (P < 0.001), excessive drinking (P < 0.001), excluding insufficient sleep (P = 0.928) and unreasonable diet (P = 0.860). This dimension had the highest proportion of health anxiety among university students. Concerning psychological anxiety, except for overpressure, there were significant gender differences in emotional instability (P = 0.013), anxiousness (P = 0.005), and depression (P = 0.037). For the appearance anxiety dimension, all three items for skin problems (P < 0.001), alopecia (P < 0.001), and obesity (P = 0.042) had significant gender differences. Thus, lifestyle, psychological and physical anxiety are major health problems faced by university students. Among them, lack of exercise (67.22%), insufficient sleep (61.41%), and emotional instability (41.33%) were the three most serious health anxiety among the total 18 health anxieties, accounting for over 40%.

### Gender Differences in Ehealth Literacy

In this study, the eHealth literacy scale consisted of three levels: functional (3 items), interactive (4 items), and critical eHealth literacy (5 items). No significant gender differences were found across these three levels (Table 3). Specifically, in functional and interactive eHealth literacy, only the second item showed a significant gender difference (P = 0.021). In critical eHealth literacy, significant gender differences were observed, excluding the tenth item (P = 0.481). Analysis of variance revealed significant differences among the means of functional, interactive, and critical eHealth literacy (P < 0.001), but no significant gender differences (P = 0.824). The mean scores indicated that critical eHealth literacy had the highest score, while functional eHealth literacy had the lowest, suggesting that university students’ reading and writing skills, as well as their understanding of basic online health information, still need improvement.

**Table 3:** Gender differences in eHealth literacy (n = 1,205) – Xinyang, Henan, China, 2024.

Items	Mn ± SD		Male	Female	P	
Functional eHealth literacy		2.79 ± 0.86	2.90 ± 1.01	2.78 ± 0.82	t	0.112
1	2.76 ± 1.00		2.87 ± 1.12	2.73 ± 0.98	t	0.068
2	2.71 ± 0.93		2.85 ± 1.08	2.69 ± 0.90	t	0.021
3	2.92 ± 1.00		2.97 ± 1.11	2.91 ± 0.97	t	0.489
Interactive eHealth literacy		3.43 ± 0.69	3.44 ± 0.73	3.43 ± 0.68	t	0.852
4	3.20 ± 0.89		3.17 ± 1.01	3.21 ± 0.86	t	0.635
5	3.41 ± 0.83		3.48 ± 0.92	3.40 ± 0.80	t	0.222
6	3.53 ± 0.86		3.55 ± 0.93	3.53 ± 0.84	t	0.686
7	3.57 ± 0.80		3.54 ± 0.86	3.58 ± 0.79	t	0.564
Critical eHealth literacy		3.65 ± 0.68	3.56 ± 0.77	3.67 ± 0.66	t	0.070
8	3.77 ± 0.81		3.65 ± 0.91	3.79 ± 0.78	t	0.037
9	3.70 ± 0.80		3.60 ± 0.85	3.73 ± 0.78	t	0.032
10	3.46 ± 0.81		3.49 ± 0.85	3.45 ± 0.80	t	0.481
11	3.63 ± 0.79		3.53 ± 0.85	3.65 ± 0.77	t	0.043
12	3.69 ± 0.80		3.55 ± 0.86	3.72 ± 0.78	t	0.009

Total		3.40 ± 0.58	3.40 ± 0.67	3.40 ± 0.56	t	0.993

Kaiser-Meyer-Olkin				0.907			
Cronbach’s Alpha				0.893			

Note. Data were expressed as mean ± SD. P-values were yielded by t-test.

### Correlations in Self-Reported Health Questionnaire Between Ehealth Literacy and Health Anxiety

To analyze the correlations between self-reported health, eHealth literacy, and health anxiety, Pearson correlation analysis was conducted. As shown in Table 4, significant positive correlations were found between eHealth literacy and self-reported health. Additionally, significant negative correlations were observed between self-reported health and the four dimensions of health anxiety: lifestyle anxiety, psychological anxiety, appearance anxiety, and physical anxiety. However, there were no significant correlations between eHealth literacy and health anxiety.

**Table 4:** Pearson correlation analysis (n = 1,205) – Xinyang, Henan, China, 2024.

Characteristics	eHealth literacy	Self-reported health
eHealth literacy	–	0.133^**^
Lifestyle anxiety	0.008	– 0.249^**^
Psychological anxiety	– 0.017	– 0.230^**^
Appearance anxiety	0.008	– 0.185^**^
Physical anxiety	– 0.033	– 0.245^**^

Note. ** indicates a significant correlation at the 0.01 level (bilateral).

### Associations Among Self-Reported Health, Ehealth Literacy and Health Anxiety

As it can be seen, Table 5 showed the associations between eHealth literacy, health anxiety and self-reported health, through ordinal logistic regression analysis. For males, eHealth literacy [OR = 0.935, (CI 95% = 0.896–0.976), P = 0.002], appearance anxiety [OR = 1.482, (CI95% = 1.049 –2.095), P = 0.026] had significant impacts on males’ self-reported health. For females, eHealth literacy [OR = 0.953, (CI95% = 0.931–0.976), P < 0.001], lifestyle anxiety [OR = 1.331, (CI95% = 1.165–1.521), P < 0.001], psychological anxiety [OR = 1.171, (CI95% = 1.043–1.314), P = 0.007], physical anxiety [OR=1.839, (CI95% = 1.442–2.346), P < 0.001] had significant impacts on females’ self-reported health.

**Table 5:** Ordinal logistic regression analysis for each gender (n = 1,205) – Xinyang, Henan, China, 2024.

Characteristics	OR (CI 95%)	P		Characteristics	OR (CI 95%)	P
Male				Female		
eHealth literacy	0.935(0.896–0.976)	0.002		eHealth literacy	0.953(0.931–0.976)	< 0.001
Lifestyle anxiety	1.199(0.915–1.572)	0.189		Lifestyle anxiety	1.331(1.165–1.521)	< 0.001
Psychological anxiety	1.240(0.968–1.589)	0.089		Psychological anxiety	1.171(1.043–1.314)	0.007
Physical anxiety	1.303(0.731–2.321)	0.369		Physical anxiety	1.839(1.442–2.346)	< 0.001
Appearance anxiety	1.482(1.049–2.095)	0.026		Appearance anxiety	1.108(0.972–1.262)	0.125
Residence (Ref: Rural)	1.677(0.924–3.044)	0.089		Residence (Ref: Rural)	1.159(0.874–1.536)	0.305
Major (Ref: Engineering)				Major (Ref: Engineering)		
Humanities and Arts	0.496(0.232–1.060)	0.070		Humanities and Arts	1.058(0.718–1.560)	0.774
Science	0.584(0.301–1.133)	0.112		Science	1.033(0.795–1.342)	0.808
Age	1.037(0.821–1.309)	0.760		Age	0.974(0.854–1.111)	0.698
Parental education level (Ref: Middle school)				Parental education level (Ref: Middle school)		
PhD/Master	9.488(0.921–97.748)	0.059		PhD/Master	0.872(0.174–4.373)	0.867
Undergraduate major/ Higher education	12.991(1.241–136.016)	0.032		Undergraduate major/ Higher education	0.905(0.181–4.518)	0.903

Note. Data are expressed as odds ratio (OR) and 95% confidence interval (CI95%) of ordinal logistic regression analysis, including explanatory variables and control variables.

## DISCUSSION

Health is a complex and multidimensional concept, making it difficult to accurately measure all dimensions of an individual’s health. In many survey studies, self-reported health is often used to collect health information from respondents. This self-reported questionnaire for university students’ health comprehensively reflects their physical and mental health and is a reliable predictive indicator of health outcomes among young people^(17)^. Health anxiety refers to excessive concern and anxiety about suffering from serious illnesses based on misunderstandings about bodily sensations or changes^(18)^. Patients with high health anxiety often exhibit an unhealthier diet and cravings for food^(19)^, making them prone to overweight and obesity^(20)^, which can lead to a series of health problems. Compared to patients without health anxiety, those with health anxiety tend to undergo more examinations and utilize more health service resources.

In this study, among the survey respondents (n = 1,205), 87.3% of university students reported experiencing multiple forms of health anxiety, with 70% facing three or more types. The severity of health anxiety among university students was ranked as follows: lifestyle anxiety, psychological anxiety, appearance anxiety, and physical anxiety. Unhealthy lifestyles have become the primary factor endangering the health of university students and contributing to mortality^(21)^. A lack of physical exercise, insufficient sleep, and excessive daytime sleepiness caused by staying up late, along with unhealthy eating habits, ultimately lead to psychological health issues and appearance anxiety, such as insomnia, alopecia, obesity, poor skin, anxiety, and excessive stress^(22,23)^. Thus, the unhealthy lifestyle behaviors of university students are the main causes of their health anxiety. If university students do not modify their behaviors through timely interventions, it may result in irreversible consequences in the future.

Self-health diagnosis and treatment through online health-seeking behaviors are among the primary methods for addressing health problems for university students. Studies have shown that university students use the Internet more frequently than any other group, which encourages them to seek health information online and promote their well-being^(24)^. However, the quality of online health information varies, requiring university students to possess adequate eHealth literacy. eHealth literacy has a significant positive impact on the quality of information and the credibility of information sources^(25)^. A higher level of eHealth literacy is associated with a greater number of information-seeking channels. University students with higher eHealth literacy possess better abilities for information acquisition, evaluation, and utilization, while those with lower eHealth literacy encounter more difficulties in searching for information^(26)^. Although studies have shown that individuals with a certain level of eHealth literacy are less likely to blindly believe that their health status is threatened when faced with an abundance of online health information, they may still experience negative emotions such as health anxiety. However, this study cannot conclusively prove that higher eHealth literacy correlates with lower health anxiety among university students. Those with high eHealth literacy may have strong abilities to query, evaluate, and apply online health information; however, excessive or improper use of this information may lead them to exaggerate or misinterpret physical symptoms, resulting in heightened health concerns and fear of illness, thereby exacerbating anxiety^(27)^.

There are several limitations to this study. First, the research employed a cross-sectional design, which cannot determine causality among the study variables. Second, only self-reported health was used to evaluate university students’ health status, which is a subjective assessment of an individual’s health. Third, this study cannot establish that lower eHealth literacy correlates with higher health anxiety among Chinese university students. The association between these two factors requires further in-depth research through various methods.

## CONCLUSION

A total of 1,205 students voluntarily participated in the survey. The severity levels of health anxiety among university students were ranked as follows: lifestyle anxiety, psychological anxiety, appearance anxiety, and physical anxiety. Significant gender differences were observed in appearance anxiety, but no significant gender differences were found in eHealth literacy. The Pearson correlation analysis and ordinal logistic regression model revealed that eHealth literacy was significantly positively associated with self-reported health, while appearance anxiety was significantly negatively associated with males’ self-reported health. Additionally, lifestyle, psychological, and physical anxiety were significantly negatively associated with females’ self-reported health. The findings suggest that lower eHealth literacy and higher levels of health anxiety are correlated with worse self-reported health. Therefore, it is necessary to develop and implement gender-based interventions to reduce health anxiety among university students in the future. In particular, university students who receive negative results regarding health anxiety from the survey will require additional health care.
